# Nitrogen dioxide – Determination of nitrogen dioxide in the workplace air using ion chromatography (IC): Air Monitoring Method – Translation of the German version from 2025

**DOI:** 10.34865/am1010244e10_3or

**Published:** 2025-09-29

**Authors:** Christian Monsé, George Dragan, Ulrich Prott, Christoph Emmel, Ralph Hebisch, Uta Lewin-Kretzschmar, Andrea Hartwig, MAK Commission

**Affiliations:** 1 Institute for Prevention and Occupational Medicine of the DGUV Institute of the Ruhr University Bochum (IPA). German Social Accident Insurance (DGUV) Bürkle de la Camp-Platz 1 44789 Bochum Germany; 2 Federal Institute for Occupational Safety and Health (BAuA) Friedrich-Henkel-Weg 1–25 44139 Dortmund Germany; 3 Berufsgenossenschaft der Bauwirtschaft (BG BAU) Am Knie 6 81241 München Germany; 4 German Social Accident Insurance, Institution for the raw materials and chemical industry, Prevention - Department of Hazardous Substances, Biological Agents and Analytical Chemistry Kurfürsten-Anlage 62 69115 Heidelberg Germany; 5 Institute of Applied Biosciences. Department of Food Chemistry and Toxicology. Karlsruhe Institute of Technology (KIT) Adenauerring 20a, Building 50.41 76131 Karlsruhe Germany; 6 Permanent Senate Commission for the Investigation of Health Hazards of Chemical Compounds in the Work Area. Deutsche Forschungsgemeinschaft, Kennedyallee 40, 53175 Bonn, Germany. Further information: Permanent Senate Commission for the Investigation of Health Hazards of Chemical Compounds in the Work Area | DFG

**Keywords:** nitrogen dioxide, air analyses, analytical method, workplace measurement, hazardous substance, ion chromatography, conductivity detection, IC, aluminium oxide, liquid desorption

## Abstract

The working group “Air Analyses” of the German Senate Commission for the Investigation of Health Hazards of Chemical Compounds in the Work Area (MAK Commission) developed and verified the presented analytical method. It is used to determine the levels of nitrogen dioxide [10102-44-2] that occur in the workplace air. The method covers concentrations in the range from one tenth up to twice the current occupational exposure limit value (OELV) of 0.95 mg/m^3^ (0.5 ml/m^3^). Samples are collected by drawing a defined volume of air through a sampling tube filled with aluminium oxide carrier material coated with triethanolamine (TEA) using a flow regulated pump at a volumetric flow rate of 1.8 l/min. The exposure during the shift is assessed with a sampling period of 2 hours and the short-term exposure with a period of 15 minutes. Nitrogen dioxide reacts with TEA with formation of TEA nitrite and TEA nitrate. Nitrite and nitrate are extracted with ultra-pure water and analysed by ion chromatography using conductivity detection. The quantitative determination is based on multiple-point calibrations with external standards. A relative limit of quantification (LOQ) of 0.009 mg/m^3^ is obtained for an air sample volume of 216 litres. As the LOQ for a sample volume of 27 litres is well below 0.95 mg/m^3^, the short-term exposure limit (STEL; excursion factor 2) can also be measured. The mean recovery is 108% and the expanded uncertainty is below 28% for a sampling period of 2 hours.

**Table:** 

**Method number**	1
**Application**	Air analysis
**Analytical principle**	Ion chromatography with conductivity detection (IC)

## 1 Characteristics of the method

**Table:** 

**Precision:**	Standard deviation (rel.):	*s* = 4–5%
	Expanded uncertainty:	*U* = 28%
	in the concentration range of 0.095–1.9 mg/m^3^ (0.05–1 ml/m^3^) and for n = 6 determinations	
**Limit of quantification:**	1.9 µg absolute	
	0.009 mg/m^3^ (0.005 ml/m^3^) for an air sample volume of 216 l and a sampling period of 2 h	
**Recovery:**	*η* = 99–115%	
**Sampling recommendations:**	Sampling period:	2 h
	Air sample volume:	216 l
	Volumetric flow rate:	1.8 l/min
	For short-term measurements:	15 min; 1.8 l/min

## 2 Description of the substance

### Nitrogen dioxide [10102-44-0]

**Fig. 1:**
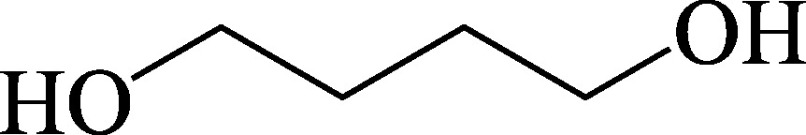
Structural formula of nitrogen dioxide

At 20 °C, nitrogen dioxide (see Figure 1, also called nitrogen(IV) oxide or nitrogen peroxide) is a reddish-brown liquid that is poisonous and has a pungent, chlorine-like odour. Nitrogen dioxide generally occurs at the workplace as a gas because it has a very high vapour pressure of 960 hPa and a low boiling point of only 21.1 °C. Nitrogen dioxide and the colourless dinitrogen tetroxide exist in an equilibrium that is dependent on pressure and temperature. In low temperature and/or high pressure conditions, as found in compressed gas cylinders, nitrogen dioxide occurs mainly as the diamagnetic dimer (IFA 2024).

Nitrogen dioxide is not combustible; however, it is a powerful oxidation agent and reacts violently or explosively with many substances. Industrially, it is synthesized in the Ostwald process by the catalytic combustion of ammonia and is subsequently hydrolysed to produce nitric acid. Mainly nitrogen dioxide, but also other nitrogen oxides form as unintended by-products of technical combustion reactions, such as those that occur in combustion engines and particularly in diesel engines. However, nitrogen oxides are released also during welding, by electric arc furnaces and during glass manufacturing (IFA 2024; RÖMPP-Redaktion and Sitzmann 2010).

An OELV of 0.95 mg/m^3^ (0.5 ml/m^3^) has been established for nitrogen dioxide. The short-term value is assigned to Peak Limitation Category I with an excursion factor of 2 (AGS 2024). The MAK value is the same as the OELV with Peak Limitation Category I with an excursion factor of 1 (DFG 2024). The substance data for nitrogen dioxide are given in Table 1.

**Tab. 1:** Substance data for nitrogen dioxide (IFA 2024)

Name	nitrogen dioxide
CAS No.	10102-44-0
Molar mass [g/mol]	46.01
Physical state at 20 °C	liquid
Density at 20 °C [g/cm^3^]	1.45
Vapour pressure at 20 °C [hPa]	960
Melting point [°C]	–11.2
Boiling point at 1013 hPa [°C]	21.1
Flash point [°C]	–
Criteria of assessment	
OELV, MAK value, Germany (AGS 2024; DFG 2024)	0.95 mg/m^3^ (0.5 ml/m^3^)
Peak limitation category (excursion factor) (AGS 2024) / (DFG 2024)	I(2) / I(1)

## 3 General principles

This analytical method is used to determine the nitrogen dioxide concentration in the workplace air in the range of one tenth to twice the currently valid OELV of 0.95 mg/m^3^ (0.5 ml/m^3^) (AGS 2024). The method is also suitable for monitoring compliance with the peak limitation category and the excursion factor of 1 (MAK value) (DFG 2024; DIN 2021 a).

Samples are taken by drawing a defined volume of air from the breathing zone through a sampling system using a pump of suitable capacity. The sampling system consists of a glass tube filled with an Al_2_O_3_ carrier material that has been impregnated with TEA. The nitrogen dioxide reacts with the TEA to form TEA nitrite and TEA nitrate, thereby binding to the sorbent (Cape 2009). The sampling tube loaded with nitrogen dioxide (TEA nitrite and TEA nitrate) is transferred to a 50-ml plastic vial, covered with ultrapure water, and then shaken. The analytical determination is carried out by IC with conductivity detection. The quantitative analysis of the nitrite and nitrate signals is performed using two separate multiple point calibrations with external standards. The amounts of both ions are added together to determine the nitrogen dioxide concentration in air.

## 4 Equipment, chemicals and solutions

### 4.1 Equipment

For the preparation of the sample carriers:

- Laboratory oven to anneal Al_2_O_3_ at 850 °C
- Crucibles or incinerating dishes made of porcelain, able to withstand temperatures up to at least 850 °C
- Disposable syringes made of polyethylene (PE) with Luer-lock connection, 10 ml (e.g. BD Discardit II, from Becton Dickinson and Company, Warwick, RI, USA)
- Rotary evaporator with temperature-controlled water bath and a 1-l rotary evaporator flask (e.g. Rotavapor R-210 with B-491 water bath, from Büchi Labortechnik GmbH, 45127 Essen, Germany)
- Vacuum pump, final vacuum at least 20 mbar (e.g. MZ 2C + AK + EK, from Vakuubrand GmbH & Co. KG, 97877 Wertheim, Germany)
- Storage containers made of glass with screw caps for the Al_2_O_3_ carrier material
- Laboratory balance, weighing range 0.1–1000 g
- 50-ml beaker and, if necessary, glass funnel for pouring the carrier material
- Glass tube, length (L) 67.2 mm, outer diameter (OD) 20 mm, inner diameter (ID) 15 mm, stopper made of polytetrafluoroethylene (PTFE) with hole and two small Viton O-rings of suitable size, two supporting sieves made of plastic, each with two Viton O-rings of suitable size (e.g. glass tube: Art. No. 777215-20, PTFE stopper: Art. No. 777217-20, small Viton O-rings: Art. No. 777218, supporting sieves: Art. No. 30003043-500, Viton O-rings: Art. No. 777213, from LABC-Labortechnik Zillger KG, 53773 Hennef, Germany) capacity: min 4.0 g, max. 4.5 g
- Caps made of PE (e.g. GPN 350/28 from Pöppelmann GmbH & Co. KG, Bakumer Str. 73, 49393 Lohne, Germany)

For sampling:

- Pump for personal and stationary sampling, suitable for a flow rate of 1.8 l/min (e.g. GilAir Plus, sold by DEHA Haan & Wittmer GmbH, 71296 Heimsheim, Germany)
- Mass flowmeter for a volumetric flow rate of 0–20 l/min (e.g. TSI Flowmeter 4146, TSI GmbH, 52068 Aachen, Germany)
- Rigid connecting tube, e.g. made of polyurethane, with an outer diameter of 8 mm
- Connecting tube to the pump, e.g. made of silicone, with an inner diameter of 8 mm

For sample preparation and the analytical determination:

- Ultrapure water system (e.g. Millipore-Q-Gradient with Elix 3UV, from Merck KGaA, 64293 Darmstadt, Germany)
- Volumetric flasks, 10 ml, 200 ml and 2000 ml (e.g. from Brand GmbH + Co. KG, 97877 Wertheim, Germany)
- Bottle-top dispenser, 10 ml or 25 ml (e.g. Dispensette III, from Brand GmbH + Co. KG, 97877 Wertheim, Germany)
- Variable piston pipettes, 10–100 μl and 100–1000 μl (e.g. Reference 2, from Eppendorf SE, 22339 Hamburg, Germany)
- Variable piston pipette, 500–5000 µl (e.g. from Brand GmbH + Co. KG, 97877 Wertheim, Germany)
- Disposable syringes made of PE with Luer-lock connection, 10 ml (e.g. BD Discardit II, from Becton Dickinson and Company, Warwick, RI, USA)
- Disposable digestion vial, 50 ml, with PE cap (e.g. DigiTube, from S-prep GmbH, 88662 Überlingen, Germany)
- Tweezers
- Anti-static device (e.g. Anti-Static Ionizer, from CEM Corporation, Matthews, NC, USA)
- Disposable cannulas, 1.2 × 40 mm (e.g. BD Microlance 3, from Becton Dickinson and Company, Warwick, RI, USA)
- Syringe filter with Luer-lock connection and PTFE membrane, Ø 25 mm, pore size 0.45 μm (e.g. Chromafil Xtra H-PTFE-45/25, Ref: 729246, from Macherey-Nagel GmbH & Co. KG, 52355 Düren, Germany)
- Autosampler vials made of polypropylene, 2.5 ml with perforated stopper (e.g. Art No. 6.2743.040 and Art No. 6.2743.070, from Metrohm Deutschland GmbH & Co. KG, 70794 Filderstadt, Germany)
- Ion chromatograph with degasser, column oven, autosampler, chemical and CO_2_ suppression and conductivity detection (e.g. 930 Compact IC Flex, from Metrohm Deutschland GmbH & Co. KG, 70794 Filderstadt, Germany)
- Anion separation column with pre-column (e.g. Metrosep A Supp 5-250/4.0, Art No. 6.1006.530 with Metrosep A Supp 5 Guard/4.0, Art No. 6.1006.500, from Metrohm Deutschland GmbH & Co. KG, 70794 Filderstadt, Germany)

### 4.2 Chemicals

- Al_2_O_3_ carrier material, 1.2 mm (e.g. Art. No. SA62240, Accu sphere, from Saint-Gobain NorPro, Stow, OH, USA)
- Triethanolamine, p.a., 99% (e.g. from Merck KGaA, 64293 Darmstadt, Germany)
- Sulfuric acid, 2.5 mol/l (5N) in aqueous solution, for suppressor regeneration (e.g. AVS TITRINORM, Art. No. 30138293, VWR International, Fontenay-sous-Bois, France)
- Sodium carbonate, anhydrous, p.a., ≥ 99.9% (e.g. Art. No. 1.06392.1000, from Merck KGaA, 64293 Darmstadt, Germany)
- Sodium bicarbonate, p.a., > 99.5% (e.g. Art. No. 1.06329.1000, from Merck KGaA, 64293 Darmstadt, Germany)
- Anion multi-element IC standard solution, fluoride (5 mg/l), chloride (10 mg/l), nitrite (15 mg/l), bromide (25 mg/l), nitrate (25 mg/l), phosphate (40 mg/l), sulfate (30 mg/l) in water (e.g. Roti Star Art. No. 2668.2, from Carl Roth GmbH + Co. KG, 76231 Karlsruhe, Germany)
- Ultrapure water (ρ ≥ 18.2 MΩ × cm at 25 °C)

### 4.3 Solutions

The following solutions were prepared with the chemicals listed in Section 4.2. The solutions are stable for at least 3 months if stored in the refrigerator at +4 ℃:

**Eluent stock solution:** (0.62 mol sodium carbonate/l and 0.069 mol sodium bicarbonate/l in water)

13.14 g of sodium carbonate and 1.15 g of sodium bicarbonate are weighed out into a volumetric flask (200 ml) and dissolved in ultrapure water. The flask is filled to the mark with ultrapure water.

**Eluent:** (3.1 mmol sodium carbonate/l and 0.35 mmol sodium bicarbonate/l in water)

10 ml of the eluent stock solution is added to a 2-l volumetric flask containing about 500 ml of ultrapure water. The volumetric flask is filled to the mark with ultrapure water and then shaken.

### 4.4 Calibration standards

Ten calibration standards are prepared with dilutions of the anion multi-element IC standard solution (see Section 4.2). For this purpose, the anion multi-element IC standard solution is dispensed by piston pipette into separate 10-ml volumetric flasks in the volumes given in Table 2 and the flasks are then filled to the mark with ultrapure water. The original, undiluted solution is the highest calibration standard “XI”.

**Tab. 2:** Pipetting scheme for the preparation of the calibration solutions and their concentrations

**Calibration standard**	**Volume of anion multi-element IC standard solution** **[ml]**	**Final volume** **[ml]**	**Concentration of nitrite** **[mg/l]**	**Concentration of nitrate** **[mg/l]**
I	0.050	10	0.0750	0.125
II	0.100	10	0.150	0.250
III	0.250	10	0.375	0.625
IV	0.500	10	0.750	1.25
V	0.750	10	1.13	1.88
VI	1.00	10	1.50	2.50
VII	2.00	10	3.00	5.00
VIII	4.00	10	6.00	10.0
IX	6.00	10	9.00	15.0
X	8.00	10	12.0	20.0
XI	10.0	10	15.0	25.0

The calibration standards can be used for 2 weeks if stored in a cool and dark place at about 4 °C.

### 4.5 Control standards

The calibration is verified by taking a measurement with the undiluted anion multi-element IC standard solution (see Section 4.4, cf. calibration standard XI). Nitrite and nitrate may not deviate by more than ± 5%.

## 5 Sampling and sample preparation

### 5.1 Preparation of the sample carriers

The sampling tubes contain an Al_2_O_3_ carrier material that has been impregnated with TEA. Before impregnation, the carrier material is first annealed in a porcelain crucible for 2 hours at 850 °C and then let cool to room temperature. After annealing, the carrier material can be stored in a sealed glass storage container and impregnated at a later time. For impregnation, 300 ml of ultrapure water is placed into a 1-l rotary evaporator flask and 10 ml of TEA is added by disposable syringe. 120 g of the annealed Al_2_O_3_ carrier material is then added. Immediately after mixing, the flask is slowly spun by rotary evaporator in a water bath heated to 65 °C at a vacuum of about 20 mbar until all the water has evaporated. This process takes about 2 hours. The pourable, impregnated Al_2_O_3_ carrier material is either transferred to a storage container (glass with cap) for storage or filled directly into sampling tubes. The impregnated carrier material can be stored for at least 3 months. Storage of the material for longer periods of time must first be assessed.

**Fig. 2:**
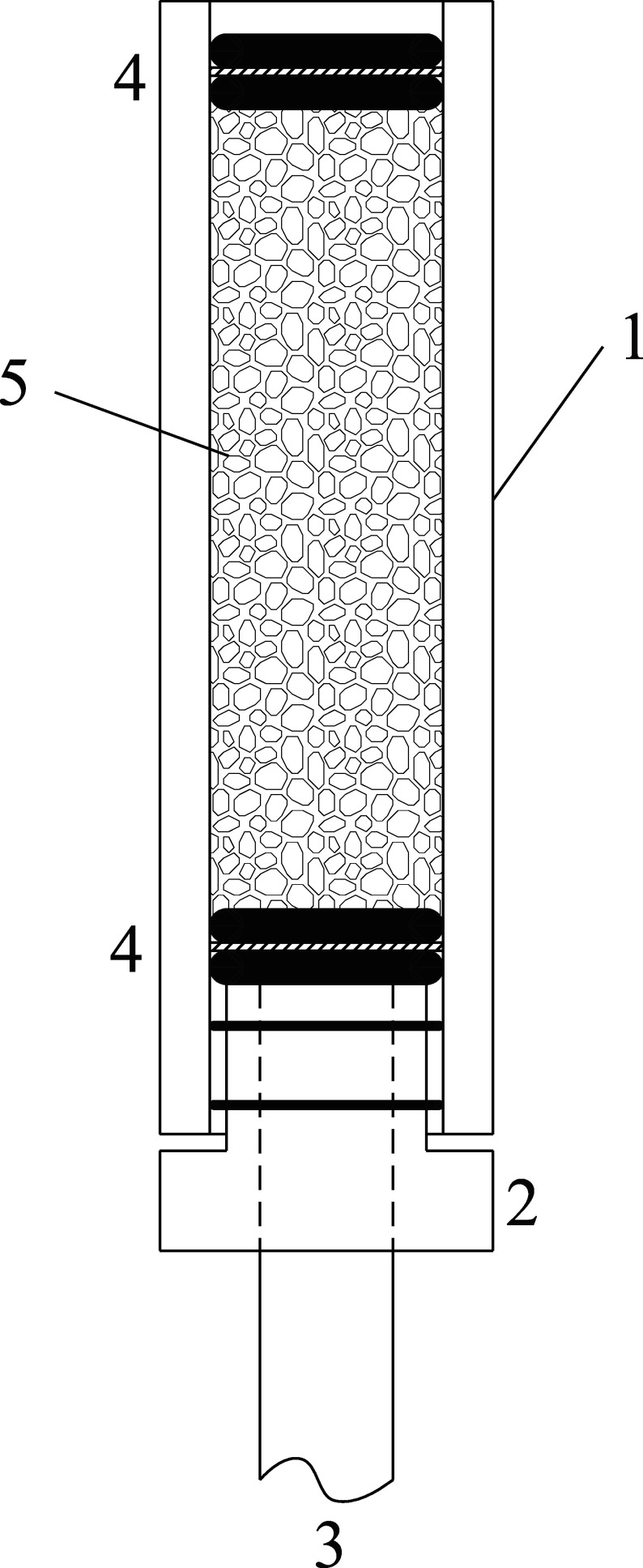
Diagram of an assembled sample carrier consisting of 1) a glass tube, 2) a PTFE stopper with two small Viton O-rings and 3) an inserted 8-mm tube, 4) plastic supporting sieves between two Viton O-rings and 5) Al_2_O_3_-carrier material impregnated with TEA

The sample carriers are prepared before beginning with sampling. For this purpose, the equipment listed in Section 4.1 is arranged as shown in Figure 2. A PTFE stopper with hole [2], which is equipped with two Viton O-rings to secure the stopper in place, is inserted into the lower end of the glass tube [1]. A plastic supporting sieve [4] is placed into [1] and secured with two Viton O-rings. 4.0 to 4.5 g per sample carrier of the impregnated Al_2_O_3_ carrier material is added to the glass tube using, for example, a 50-ml beaker and, if necessary, a funnel. The upper end is sealed with two O-rings and a second supporting sieve [4]. The sample carrier is sealed with caps made of PE until sampling.

### 5.2 Sampling

Samples are collected using stationary or personal sampling procedures. The personal samples are collected vertically within the breathing zone. The inlet of the sampling head must remain unobstructed during sampling.

The caps are removed from the glass tube immediately before sampling. A connecting tube with an outer diameter of 8 mm [3] is inserted into the hole of [2]. This tube is connected to the sampling pump via another connecting tube with an inner diameter of 8 mm. Sample air is drawn through the sampling system using a flow-regulated pump at a volumetric flow rate of 1.8 l/min. Sampling for 2 hours is equivalent to an air sample volume of 216 l. The main parameters for determining concentrations in air (air sample volume, temperature, air pressure and relative humidity) are documented in a sampling record.

After sampling, the flow rate must be tested for constancy. If the deviation from the adjusted volumetric flow rate is greater than ± 5%, the sample should be discarded (DIN 2023). The tubes are tightly sealed with caps and transported to the laboratory. The samples should be processed within 7 days.

Each series of samples must include a field blank. The only difference in the handling of this sample and the analytical samples is that an air sample is not drawn through the filter. The field blank is stored and processed in the same manner as the samples.

### 5.3 Sample preparation

The cap is removed from the inlet (upper end) of the glass tube, tweezers are used to remove the supporting sieve and the Al_2_O_3_ carrier material is transferred to a 50-ml plastic vial (DigiTube).

The Al_2_O_3_ beads may be electrostatically charged. An anti-static device reduces the electrostatic charge, facilitating the transfer of the beads. 17 ml of ultrapure water is added with a dispenser. The tube is closed with a cap and lightly shaken by hand for about a minute. The tube is let stand for 1 hour and then briefly shaken again by hand. The tube is let stand for another hour to allow the suspended solid to precipitate.

A 10-ml disposable syringe with an attached disposable cannula is used to draw out about 4 ml of the supernatant fluid. The cannula is removed and replaced by a syringe filter. About 0.5 ml of the liquid is passed through the filter and discarded. Another about 2.5 ml is passed through the same filter into an autosampler vial. The autosampler vial is sealed and placed into the autosampler for IC analysis. The sample vial with the remaining fluid is sealed and stored in the refrigerator as a reserve sample.

*Note: Very fine particles are abraded from the carrier material during desorption. These are very difficult to separate from the solution. The syringe filters become clogged very quickly and filtration therefore requires considerable force.*

## 6 Operating conditions

**Table:** 

**Apparatus:**	Ion chromatography system (IC) with degasser, column oven, autosampler, chemical and CO_2_ suppression
**Pre-column:**	Metrosep A Supp 5 Guard/4.0, ID 4 mm, L 5 mm
**Separation column:**	Metrosep A Supp 5-250/4.0, ID 4 mm, L 250 mm
**Column temperature:**	45 °C
**Detector:**	conductivity detector
**Eluent:**	3.1 mmol sodium carbonate/l and 0.35 mmol sodium bicarbonate/l, isocratic
**Flow rate:**	0.7 ml/min
**Injection volume:**	20 µl
**Run time:**	42 min

Under the given conditions, the retention time of nitrite was about 11.4 minutes and that of nitrate about 15.0 minutes.

## 7 Analytical determination

The analytical determination is performed by injecting 20 μl of each of the samples prepared as described in Section 5.3 into the ion chromatograph system and analysing the samples under the conditions given in Section 6. The concentrations of the two analytes nitrate and nitrite are determined based on two separate calibration curves (see Section 8).

If the resulting concentrations lie above the calibration range, suitable dilutions are prepared with the eluent and then analysed. The prepared field blank and lab blank are analysed in the same manner as the analytical samples.

## 8 Calibration

### External calibration

The calibration standards described in Section 4.4 are analysed as described in Sections 6 and 7 to derive the calibration functions. The resulting peak areas are plotted against the respective concentrations. The calibration functions are linear in the investigated concentration range.

The control standard described in Section 4.5 is analysed every working day to check the calibration functions. A new calibration must be performed if the analytical conditions change or the results of the quality control indicate that this is necessary.

## 9 Calculation of the analytical result

The nitrogen dioxide concentration in the workplace air is calculated by determining the concentrations of the nitrite and nitrate ions in the solution. The data analysis programme and the calibration function obtained for each ion are used to determine the respective concentrations *c(NO_2_^–^) *and* c(NO_3_^–^)* in mg/l based on the peak areas of the signals of nitrate and nitrite. The amount of nitrogen dioxide collected from each sample carrier is calculated according to Equation 1, taking into consideration the blank values for both ions and the desorption volume.



(1)

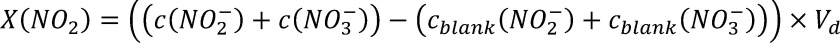




where:

**Table:** 

*X(NO_2_)*	is the mass of nitrogen dioxide per sample carrier in mg
*c(NO_2_^–^)*	is the nitrite concentration in the measurement solution in mg/l
*c(NO_3_^–^)*	is the nitrate concentration in the measurement solution in mg/l
*c_blank_(NO_2_^–^)*	is the nitrite concentration in the field blank (mean) in mg/l
*c_blank_(NO_3_^–^)*	is the nitrate concentration in the field blank (mean) in mg/l
*V_d_*	is the volume of the eluate in litres (in this case 0.017 l)

The nitrogen dioxide concentration in the workplace air is calculated according to Equation 2, taking into consideration the air sample volume and recovery. If a recovery of 100 ± 5% is determined in the range from one tenth to twice the limit value, Equation 2 does not require correction.



(2)

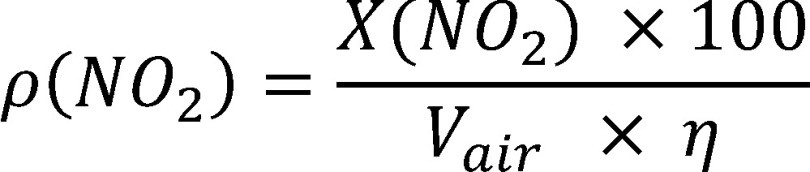




where:

**Table:** 

*ρ(NO_2_)*	is the mass concentration of nitrogen dioxide in the air sample in mg/m^3^
*V_air_*	is the air sample volume in m^3^ (calculated from the volumetric flow rate and the sampling period, in this case 0.216 m^3^ after sampling for 2 hours)
*ƞ*	recovery in %

These values are extrapolated to 20 °C and 1013 hPa according to Equation 3:



(3)

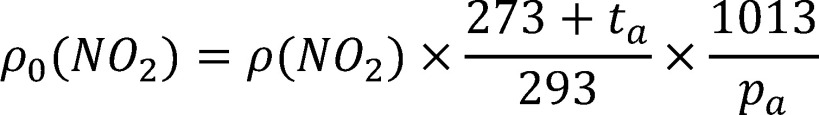




where:

**Table:** 

*ρ(NO_2_)*	is the mass concentration of the substance in the air sample in mg/m^3^ at *t_a_* and *p_a_*
*ρ_0_(NO_2_)*	is the mass concentration of the substance in mg/m^3^ at 20 °C and 1013 hPa
*t_a_*	is the temperature during sampling in °C
*p_a_*	is the air pressure during sampling in hPa

## 10 Reliability of the method

The characteristics of the method were determined according to the standards DIN EN 482 (DIN 2021 a), DIN EN ISO 22065 (DIN 2021 b) and DIN 32645 (DIN 2008). The method was fully validated.

To validate the method, the sample carriers were loaded with test gas. For this purpose, a controlled atmosphere with defined concentrations was generated in an exposure laboratory and samples were then taken. The experimental set-up for the generation of the controlled atmosphere is described in detail in Monsé et al. (2020). The laboratory did not have the technical capabilities to perform tests with a concentration of 0.095 mg/m^3^ (0.05 ml/m^3^), which is equivalent to one tenth of the OELV. Instead, test gas with double the concentrations (0.2 × OELV, 2 × OELV, 4 × OELV) was drawn through the sample carriers for 1 hour (half of the recommended measurement period) at a volumetric flow rate of 1.8 l/min. After extrapolation to a sampling period of 2 hours and an air sample volume of 216 l, these loads are equivalent to nitrogen dioxide concentrations in air of 0.095 mg/m^3^, 0.95 mg/m^3^ and 1.9 mg/m^3^ (0.050 ml/m^3^, 0.50 ml/m^3^ and 1.0 ml/m^3^), which are equivalent to one tenth of the OELV, the OELV and twice the OELV. The sampling tubes were then prepared according to the steps described in Section 5.3 and the sample solutions were analysed after preparation as described in Sections 6 and 7.

### 10.1 Precision, recovery and expanded uncertainty

To determine precision and expanded uncertainty, sets of 6 sample carriers per test concentration were prepared and then analysed as described in Section 10.

The data obtained for precision, recovery and expanded uncertainty are shown in Table 3. The precision and recovery data are based on the amount of nitrogen dioxide that was calculated by adding together the amounts of nitrate and nitrite.

**Tab. 3:** Standard deviation (rel.) and expanded uncertainty *U* for n = 6 determinations

**Concentration^a)^** **[mg NO_2_/m^3^] ([ml NO_2_/m^3^])**	**Recovery** **[%]**	**Standard deviation (rel.)** **[%]**	**Expanded uncertainty *U*** **[%]**
0.095 (0.050)	99.4	5.0	28.5
0.95 (0.50)	115.1	3.6	28.3
1.9 (1.0)	104.8	3.9	28.4

^a)^ The concentration is obtained for a sampling period of 2 hours at a volumetric flow rate of 1.8 l/min.

The expanded uncertainty was determined by estimating all relevant influencing parameters. The two main sources of uncertainty in the measurement results are uncertainties in the sampling procedure and in the analytical procedure. For this purpose, the Excel tool provided by IFA for the calculation of expanded uncertainty was applied to the calibration curves taking into account an estimated uncertainty of 2% (DIN 2021 b; IFA n.d.).

The combined, concentration-dependent uncertainties for the entire method were calculated by combining the contributions from all sources of uncertainty. The percentages listed in Table 3 for the expanded uncertainty for the entire method were obtained by multiplying the values with the expansion factor k = 2.

An expanded uncertainty of at most 29.0% is calculated for a sampling period of 15 minutes (short-term value).

### 10.2 Influence of humidity

The influence of humidity was investigated using concentrations equivalent to one tenth to twice the OELV at a relative humidity of about 35% and 65%. For this purpose, 4 sample carriers per concentration and set level of humidity were loaded with test gas as described in Section 10. The sample carriers were prepared and analysed as described in Sections 5.2, 6 and 7. The relative humidity was not found to influence recovery.

### 10.3 Limit of quantification

The limits of quantification for nitrite and nitrate were determined according to DIN 32645 (DIN 2008), each based on an equidistant 10-point calibration in the lower concentration range.

The standards for the 10-point calibration were prepared with dilutions of the anion multi-element IC standard solution. For this purpose, the standard solution was added to 10-ml volumetric flasks containing 5 ml of eluent in the volumes given in Table 4. The flasks were then filled to the mark with eluent and shaken. Table 4 shows the concentrations of the 10 calibration standards.

**Tab. 4:** Pipetting scheme for the preparation of the 10 calibration standards in the lower concentration range for determining the limit of quantification

**Calibration standard**	**Volume of anion multi-element IC standard solution** **[µl]**	**Final volume** **[ml]**	**Concentration nitrite** **[mg/l]**	**Concentration nitrate** **[mg/l]**
I	100	10	0.150	0.250
II	200	10	0.300	0.500
III	300	10	0.451	0.750
IV	400	10	0.601	1.00
V	500	10	0.751	1.25
VI	600	10	0.901	1.50
VII	700	10	1.05	1.75
VIII	800	10	1.20	2.00
IX	900	10	1.35	2.25
X	1000	10	1.50	2.50

At a 95% confidence interval, the absolute limit of quantification was 1.9 µg of NO_2_ per sample carrier. This is equivalent to 0.009 mg NO_2_/m^3^ (0.005 ml/m^3^) for an air sample volume of 216 litres (1.8 l/min and sampling for 2 h) and an eluent volume of 17 ml.

### 10.4 Capacity of the sampling system

To determine the breakthrough behaviour of the sampling system used for testing, test gas with a concentration of 3.8 mg/m^3^ (2.0 ml/m^3^) was generated in an exposure laboratory and then drawn through the sample carrier at a volumetric flow rate of 1.8 l/min. Breakthrough was determined by analysing the air drawn through the sampling system using online chemical ionization mass spectrometry (AirSense, from MS4-Analysentechnik GmbH, 35519 Rockenberg, Germany). The signal was not found to have increased even after 4 hours. Therefore, the sampling system is suitable also for sampling for 4 hours at 4 times the OELV or MAK value.

### 10.5 Storage stability

The storage stability was determined by loading sets of 8 sample carriers with concentrations equivalent to one tenth and twice the OELV as described in Section 10. Four sample carriers per concentration were prepared and analysed directly as described in Sections 5, 6 and 7. The contents of the other four sampling tubes per concentration were transferred to 50-ml vials (DigiTube). 17 ml of ultrapure water was added, the vials were sealed airtight and stored with the seals intact for 4 weeks in the refrigerator at about 4 °C. Extracts were then prepared and analysed as described in Sections 5, 6 and 7.

The mean recovery was 92.0% after storage for 4 weeks in the refrigerator. The decreased recovery after longer storage periods must therefore be taken into account when calculating the analytical results.

### 10.6 Selectivity and interference

**Fig. 3:**
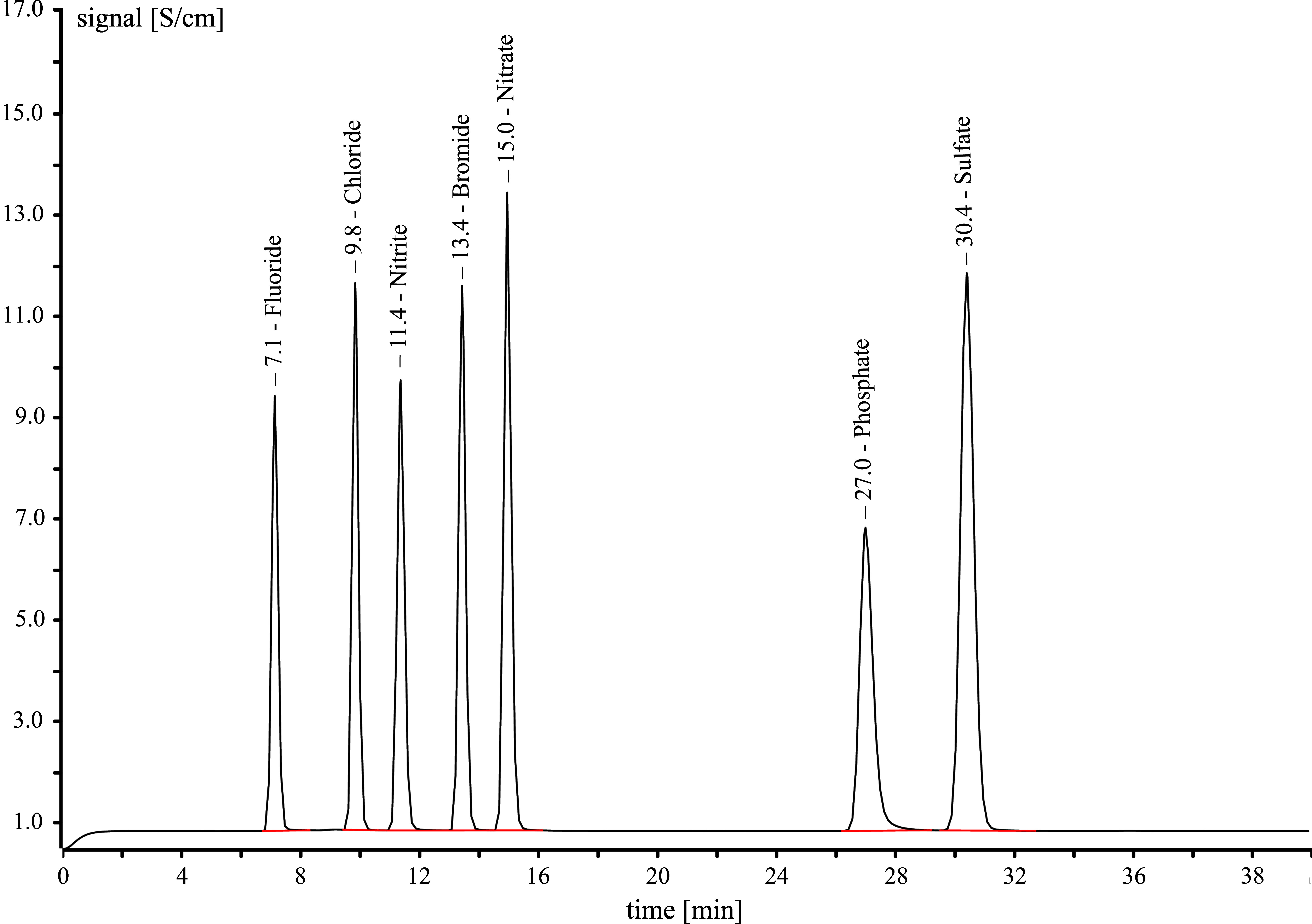
Example of a chromatogram showing the ion chromatographic separation of nitrite and nitrate from the common anions fluoride, chloride, bromide, phosphate and sulfate

The IC analytical method is specific and robust under the specified working conditions. No interference was detected. It is possible to separate nitrite, nitrate and other anions chromatographically (see Figure 3).

Not only the intended reaction between nitrogen dioxide and TEA may occur; other molecules may also react with TEA to form and release nitrite and nitrate ions. The most important sources of possible positive interference, apart from the reaction of nitrogen monoxide with ozone during sampling, are peroxyacetyl nitrate [2278-22-0], nitrous acid [7782-77-6] (Cape 2009) and nitric acid [7697-37-2].

Furthermore, nitrite and nitrate in particle form may be collected during sampling, leading to false positive results. If there is a possibility that the particulate salts of nitrous acid and nitric acid may be present, a suitable filter (e.g. PTFE) in a filter holder should be connected to the sampling tube by a length of silicone tubing with as little dead volume as possible. The filter, which is connected upstream, is not included in the analysis. The additional loss of pressure must be taken into account when adjusting the sampling pump.

## 11 Discussion

The described method is used to determine nitrogen dioxide in the workplace air in a concentration range from one tenth to twice the currently valid OELV or MAK value of 0.95 mg/m^3^ (0.5 ml/m^3^). The method is also suitable for monitoring compliance with the short-term value.
